# Genetically engineered minipigs model the major clinical features of human neurofibromatosis type 1

**DOI:** 10.1038/s42003-018-0163-y

**Published:** 2018-10-02

**Authors:** Sara H. Isakson, Anthony E. Rizzardi, Alexander W. Coutts, Daniel F. Carlson, Mark N. Kirstein, James Fisher, Jeremie Vitte, Kyle B. Williams, G. Elizabeth Pluhar, Sonika Dahiya, Brigitte C. Widemann, Eva Dombi, Tilat Rizvi, Nancy Ratner, Ludwine Messiaen, Anat O. Stemmer-Rachamimov, Scott C. Fahrenkrug, David H. Gutmann, Marco Giovannini, Christopher L. Moertel, David A. Largaespada, Adrienne L. Watson

**Affiliations:** 1 Masonic Cancer Center, University of Minnesota, Room 3-129, Cancer Cardiovascular Research Building, 2231 6th Street SE, Minneapolis, MN 55455 USA; 2 Recombinetics Inc., 1246 University Avenue W., Suite 301, St. Paul, MN 55104 USA; 3 Department of Experimental and Clinical Pharmacology, College of Pharmacy, University of Minnesota, Room 459, 717 Delaware Street SE, Minneapolis, MN 55414 USA; 4 Department of Head and Neck Surgery, David Geffen School of Medicine at UCLA and Jonsson Comprehensive Cancer Center (JCCC), University of California Los Angeles, 675 Charles E Young Drive S, MRL Room 2240, Los Angeles, CA 90095 USA; 5 Department of Veterinary Clinical Sciences, College of Veterinary Medicine, University of Minnesota, 1365 Gortner Avenue, St. Paul, MN 55108 USA; 6 Division of Neuropathology, Department of Pathology and Immunology, Washington University School of Medicine, 660S. Euclid Avenue, CB 8118, St. Louis, MO 63110 USA; 7 Pediatric Oncology Branch, Center for Cancer Research, National Cancer Institute, CRC 1-5750, 10 Center Drive, Bethesda, MD 20892 USA; 8 Division of Experimental Hematology and Cancer Biology, Department of Pediatrics, Cincinnati Children’s Hospital, University of Cincinnati, 3333 Burnet Avenue, ML 7013, Cincinnati, OH 45229 USA; 9 Medical Genomics Laboratory, Department of Genetics, University of Alabama at Birmingham, Kaul Building, 720 20th Street South, Birmingham, AL 35294 USA; 10 Department of Pathology, Massachusetts General Hospital, Warren Building, Room 333A, 55 Fruit Street, Boston, MA 02114 USA; 11 Department of Neurology, Washington University School of Medicine, Box 8111, 660S. Euclid Avenue, St. Louis, MO 63110 USA; 12 Department of Pediatrics, University of Minnesota, Room 3-129, Cancer Cardiovascular Research Building, 2231 6th Street SE, Minneapolis, MN 55455 USA

## Abstract

Neurofibromatosis Type 1 (NF1) is a genetic disease caused by mutations in *Neurofibromin 1* (*NF1*). NF1 patients present with a variety of clinical manifestations and are predisposed to cancer development. Many NF1 animal models have been developed, yet none display the spectrum of disease seen in patients and the translational impact of these models has been limited. We describe a minipig model that exhibits clinical hallmarks of NF1, including café au lait macules, neurofibromas, and optic pathway glioma. Spontaneous loss of heterozygosity is observed in this model, a phenomenon also described in NF1 patients. Oral administration of a mitogen-activated protein kinase/extracellular signal-regulated kinase inhibitor suppresses Ras signaling. To our knowledge, this model provides an unprecedented opportunity to study the complex biology and natural history of NF1 and could prove indispensable for development of imaging methods, biomarkers, and evaluation of safety and efficacy of NF1-targeted therapies.

## Abstract (web summary)

Isakson et al. report a genetically engineered minipig model of Neurofibromatosis Type 1 (NF1) that exhibits clinical hallmarks of the disease, including neurofibromas and optic pathway glioma. This model may expedite the development of imaging methods, biomarkers, and therapies for NF1 patients.

## Introduction

NF1 is a prevalent genetic disorder, occurring in one in every 3000 children born, with over two million cases worldwide^1^. NF1 patients are born with mutations in one copy of the *NF1* tumor suppressor gene, a negative regulator of the Ras signaling pathway. Over time, the remaining wild-type allele of *NF1* may be lost in a rare cell, leading to abnormal cell growth and division in multiple body systems. For instance, loss of heterozygosity (LOH) in the melanocyte lineage results in cafe au lait macules (CALMs), hyperpigmented patches of skin present in nearly all patients, and LOH in the Schwann cell lineage leads to the development of neurofibromas^1–5^. NF1 patients are predisposed to other tumors, including malignant peripheral nerve sheath tumors, optic pathway gliomas (OPGs), astrocytomas, and juvenile myelomonocytic leukemia^1,6–9^. While there has been considerable effort to develop targeted therapies for these tumors, few treatments show clinical efficacy^10–16^. In addition to tumor predisposition, NF1 patients also commonly suffer from skeletal abnormalities, scoliosis, short stature, learning disabilities, hypertension, and epilepsy^1,17–19^.

Many murine models of NF1 have been developed, yet none fully recapitulates the disease spectrum seen in NF1 patients^20,21^. The first mouse model of NF1 was a traditional germ line heterozygous knockout mouse, which developed some of the less common NF1-associated tumors, however, did not develop neurofibromas or other characteristic symptoms of NF1^22,23^. More complex mouse models have since been developed to replicate the more common features of NF1. For example, the Cre-lox system was employed to generate mice with bi-allelic loss of *Nf1* in a specific cell lineage (e.g., astrocyte, Schwann cell), and double mutant *Nf1/Tp53* mice were developed to study malignant peripheral nerve sheath tumors and astrocytomas. While these models have improved our understanding of NF1-associated tumorigenesis, each has major limitations and none display the complexity of disease observed in NF1 patients^10,24–26^. More importantly, preclinical studies in mice are often not predictive of drug efficacy in the humans^27,28^.

Large animal models that better approximate human physiology and anatomy are essential to translating discoveries from murine models into clinical therapies. In contrast to rodents, swine (*Sus scrofa*) are highly similar to humans in regard to their nervous, integumentary, and cardiovascular systems^29^. The anatomical, biochemical, and cellular components of porcine nerves are comparable to humans, making the study of NF1-associated nerve hyperplasia and neurofibroma development both feasible and applicable^29^. The porcine eye has a pigmented iris and retinal layers with a similar thickness to humans, allowing the use of imaging modalities to identify Lisch nodules and study OPG pathogenesis in swine^29,30^. Porcine skin is similar to humans in structure, thickness, hair follicle content, vascular anatomy, and collagen fiber arrangement, making these animals ideal for studying the development of CALMs and dermal neurofibromas^29,30^. NF1-associated hypertension is easily modeled in the pig, which has long been used as a model for human cardiovascular disease due to their nearly identical cardiac anatomy and physiology^29^. Finally, cytochrome P450 enzymes, which function to metabolize drugs in humans, are genetically very similar to those in the pig, making them a valuable model for predicting drug efficacy and toxicology in humans^30^.

Recent technological advances in precision gene targeting and somatic cell nuclear transfer have given researchers the ability to produce genetically modified swine carrying exact disease alleles found in human patients. Genetically engineered swine as large animal biomedical models open a vast array of new opportunities to study disease and develop novel, safe, and effective therapies. Here, we describe the development and characterization of an Ossabaw minipig model of NF1. The Ossabaw minipig is derived from a population of feral pigs on Ossabaw Island, Georgia, United States. They have a life expectancy of up to 15 years and reach puberty at 5–6 months, making them an ideal animal model for studying disease features that occur in both pediatric and adult populations. We estimate that a 6-month-old Ossabaw minipig is equivalent to a pubescent human and each year of life thereafter corresponds to a decade in the human. Therefore, a 1-year-old Ossabaw minipig would correspond to a 20-year-old human and a 2-year-old Ossabaw minipig would correspond to a 30-year old human. The long lifespan of the Ossabaw minipig allows for longitudinal and natural history studies of human diseases. Further, the Ossabaw minipig grows to a size of 180–220 pounds, roughly the size of an adult human, making it possible to perform advanced imaging studies on instruments designed for humans.

We have developed an NF1 minipig that recapitulates the diverse phenotypes seen in NF1 patients, including the development of CALMs, neurofibromas, and OPG. NF1 minipigs exhibit spontaneous and cell-type-specific LOH, a critical step for both CALM and neurofibroma development in NF1 patients and a hallmark of NF1 that has not been observed in rodent models. We demonstrate that NF1 minipigs can be dosed orally with a small-molecule inhibitor, currently in clinical development for NF1, which leads to a targeted reduction in Ras signaling. This NF1 minipig model provides a unique opportunity to study the complex biology and natural history of NF1 and could prove indispensable for preclinical evaluation of NF1-targeted therapies as well as development of imaging methods and diagnostic biomarkers.

## Results

### Generation of an NF1 minipig by gene editing

**Fig. 1:**
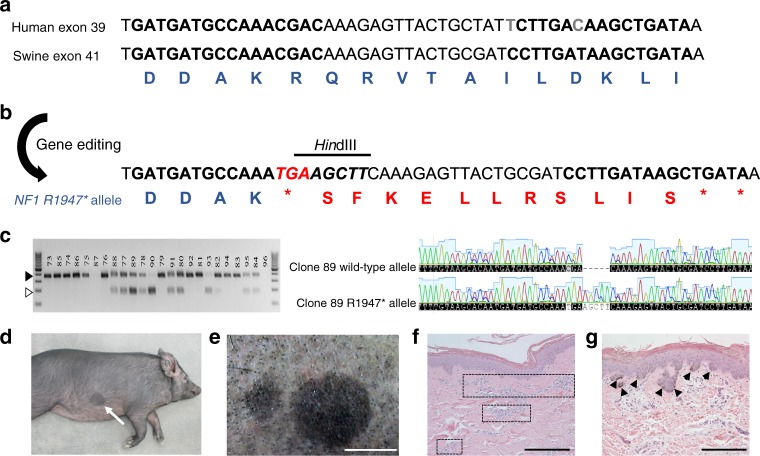
Development of NF1 minipigs. **a** Human exon 39 and swine exon 41 of the *NF1* gene show 100% amino acid homology. A pair of TALENs was designed to bind swine *NF1* exon 41 in the region of R1947. The entire exon is not shown; gray letters, differences in nucleotide sequences; bold letters, TALEN-binding sites; blue letters, amino acid sequence. **b** The *NF1*^R1947*^ allele was engineered into the swine genome using homologous recombination (HR) of a 90mer HDR oligonucleotide containing a *Hin*dIII RFLP site to allow for facile analysis of HR-positive cells. Bold letters, TALEN-binding sites; red italicized nucleotides, novel stop codon (R1947*); black italicized nucleotides, added nucleotides; blue letters, amino acids; red letters, amino acid code resulting from frameshift. **c** Minipig embryonic fibroblasts were transfected with TALENs and HDR oligonucleotides and individual cells were isolated and grown as single cell-derived colonies. These colonies were assayed for incorporation of the RFLP site by *Hin*dIII restriction enzyme digest followed by gel electrophoresis. The closed arrowhead denotes the wild-type allele and the open arrowhead denotes the RFLP allele. Several clones were TOPO cloned and sequenced to confirm the presence of both the wild-type allele and the *NF1*^R1947*^ allele (clone 89 is shown as an example). Three to five sequence-confirmed *NF1*^R1947*/+^ clones were pooled and underwent chromatin transfer to produce F0 NF1 male minipigs that were subsequently bred to wild-type females to produce F1 minipigs. **d** A representative example of a CALM (white arrow) seen in an NF1 minipig at 5 months of age. **e** An example of multiple CALMs seen in an NF1 minipig at 16 months of age. Scale bar, 2 cm. **f** H&E staining of adjacent normal NF1 minipig skin and wild-type minipig skin (shown here) shows vascular beds (squares) and melanin but no melanin deposits. Scale bar, 200 µM. **g** H&E staining of CALMs shows melanin deposits in the basal layer of the epidermis (black arrowheads). Scale bar, 200 µM

To generate a minipig model of NF1, we mimicked a recurrent nonsense mutation p.Arg1947*(R1947*) identified in 62 of 8100 (±8) unrelated and symptomatic NF1 patients (Supplementary Table 1)^31^. This mutation has also been described in several other studies^32–36^. *NF1*^*R1947*^ lies within exon 41 of the swine *NF1* gene, which shares 100% amino acid identity with human exon 39 (Fig. 1a)^37^. Transcription activator-like effector nucleases (TALENs) flanking *NF1*^*R1947*^ were transfected into fetal Ossabaw minipig fibroblasts with a homology directed repair (HDR) oligonucleotide containing the R1947* mutation and a *Hin*dIII restriction fragment length polymorphism (RFLP) site (Fig. 1b, Supplementary Table 2). Colonies derived from single cells were isolated and genotyped for the *NF1*^R1947*^ mutation (Fig. 1c). Heterozygous clones were subjected to chromatin transfer resulting in two viable pregnancies and eight F0 male piglets. *NF1*^R1947*/+^ (NF1) F0 minipigs were sequence validated, subsequently bred to wild-type sows and exhibited germ line transmission of the mutant *NF1* allele with Mendelian frequency. A total of 105 F1 piglets were produced from the first 15 litters: 54% (57) wild type and 46% (48) NF1, with no evidence of reduced fitness in NF1 minipigs. Germ line transmission of the mutant *NF1* allele was also demonstrated by breeding NF1 females to wild-type males.

### NF1 minipigs display CALMs and other skin abnormalities

From birth, all NF1 F0 and F1 minipigs displayed multiple flat, dark brown skin patches resembling NF1-associated CALMs, one of the most common phenotypes in NF1 patients (Fig 1d, e, Table 1). Histologically, CALMs showed hyperpigmentation of the basal layer of the epidermis, as seen in humans (Fig. 1f, g). One hundred percent of NF1 minipigs harbored six or more CALMs over 5 mm pre-puberty or 15 mm post-puberty, as stipulated in the National Institute of Health (NIH) diagnostic criterion for NF1^38^. CALMs observed in NF1 minipigs increased in size and number with age. A subset of NF1 minipigs exhibited other abnormalities described in NF1 patients, including freckling (30.8%, 4/13 animals) and congenital hypopigmentation of the hair, with no underlying melanin changes in the skin (23.1%, 3/13 animals) (Supplementary Figure 1, Table 1)^1,4,5^. In contrast, no wild-type minipigs showed CALMs or evidence of pigmentation defects (*N* > 50).

**Table 1:** Summary of NF1-related phenotypes seen in NF1 minipigs that underwent imaging

Animal ID	Generation	Sex	Age	Imaging	CALM	Freckling	Lisch nodules	Hypopigmentation	Neurofibroma	OPG	Tibial diaphysis narrowing
1728	F0	M	5.3 months	MRI, CT, X-ray	Present	Absent	N/A	Absent	Absent	N/A	Bilateral
1729	F0	M	19.8 months	MRI, CT, X-ray	Present	Absent	Absent	Absent	Several in armpit region	N/A	Absent
1730	F0	M	16.7 months	MRI, CT, X-ray	Present	Absent	Present	Present	Absent	N/A	Absent
1734	F0	M	3.7 months	MRI, CT, X-ray	Present	Absent	N/A	Absent	Absent	N/A	Bilateral
1735	F0	M	19.8 months	MRI, CT, X-ray	Present	Absent	Present	Absent	Several in armpit region	N/A	Absent
1228	F1	F	9.0 months	MRI, CT, X-ray	Present	Absent	N/A	Absent	Absent	Present	Absent
1229	F1	M	16.1 months	MRI, CT, X-ray	Present	Absent	N/A	Absent	Several in armpit region	Absent	Absent
									3 discrete tumors		
1232	F1	M	15.7 months	MRI, CT, X-ray	Present	Present	N/A	Present	Several in armpit region	Absent	Absent
									5 discrete tumors		
1233	F1	M	8.9 months	MRI, CT, X-ray	Present	Present	N/A	Absent	Absent	Absent	Absent
1236	F1	F	9.0 months	MRI, CT, X-ray	Present	Present	N/A	Present	Absent	Absent	Absent
1342	F1	F	8.8 months	MRI, CT, X-ray	Present	Absent	N/A	Absent	Absent	Absent	Absent
1346	F1	M	9.9 months	X-ray	Present	Present	N/A	Absent	Absent	N/A	Absent
1359	F1	F	9.9 months	MRI, CT, X-ray	Present	Absent	N/A	Absent	1 discrete tumor	Absent	Absent
Prevalence of phenotype					13/13	4/13	2/3	3/13	5/13	1/7	2/13

*N/A* not assessed

### NF1 minipigs develop neurofibromas

**Fig. 2:**
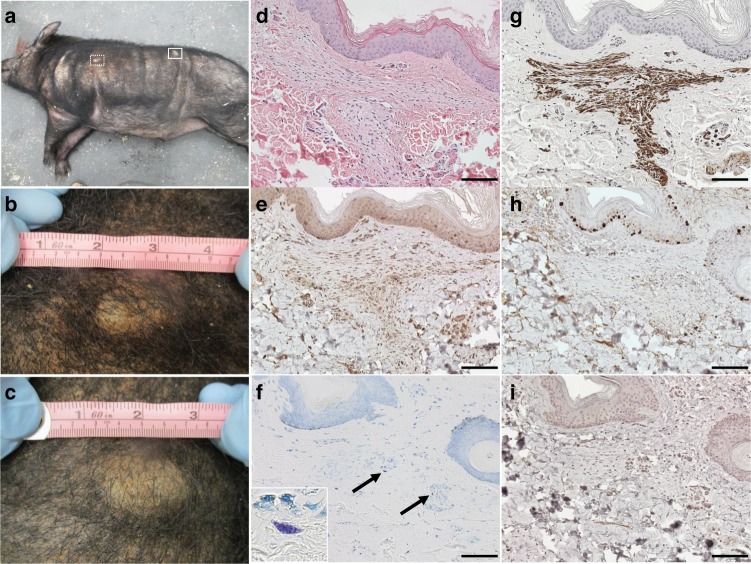
NF1 minipigs develop neurofibromas. **a** An example of an NF1 minipig harboring two dermal masses on its left side. The mass denoted by the white dotted box is enlarged in **b** and the mass denoted by the solid white box is enlarged in **c**. **b** The mass on the shoulder measured 3.5 cm in diameter. **c** The mass on the flank measured 4.2 cm in diameter. **d** H&E staining of a representative mass showing regions of hypercellularity. **e** Hypercellular regions stain positive for GFAP, a marker of Schwann cells. **f** Mast cell infiltration is shown by Toluidine blue metachromasia (purple). **g** Hypercellular regions stain positive for S100β, a marker of Schwann cells. **h** Hypercellular region showing minimal proliferation by Ki67 staining. **i** Mast cell infiltration is shown by c-Kit staining. Scale bars, 100 µM

Neurofibromas are benign peripheral nerve sheath tumors composed of multiple cell types including Schwann cells, fibroblasts, and mast cells^39^. These tumors are variable in size and number and represent a major source of pain and disfigurement in NF1 patients. Superficial tumors resembling neurofibromas were noted in 40% (2/5) of the NF1 F0 animals and 37.5% (3/8) of the NF1 F1 minipigs by 4 months of age (Table 1). Tumors were discrete and ranged in number from one to six per animal and in size from 1.8 to 6.0 centimeters in diameter (Fig. 2a–c). Hematoxylin and eosin (H&E) staining revealed areas of hypercellularity, confirmed to be clusters of Schwann cells by S100β and glial fibrillary acidic protein (GFAP) staining, surrounded by dense collagen and sparse fibroblasts (Fig. 2d, e, g). These tumors had a low Ki67 proliferative index and showed mast cell infiltration (Fig. 2f, h, i). Control immunohistochemistry was performed on sciatic nerve and intestinal tissue (Supplementary Figure 2). These data show that the tumors seen in NF1 minipigs share classic features of human neurofibromas. The skin-related abnormalities seen in NF1 minipigs closely resemble those seen in NF1 patients, and this model meets the NIH criteria for both CALMs and neurofibromas used to diagnose NF1 patients.

### NF1 minipigs develop OPG

**Fig. 3:**
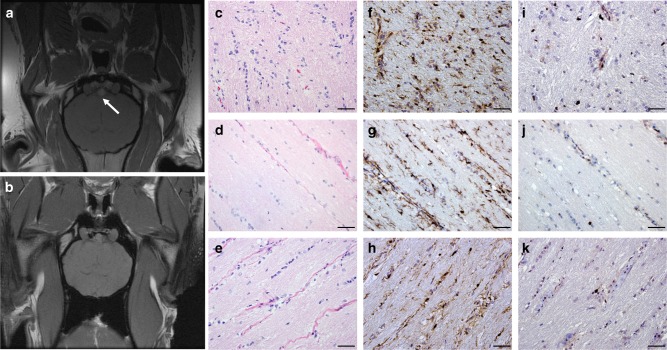
NF1 minipigs develop optic pathway glioma-like lesions. **a** Axial T1-weighted MRI demonstrates a lesion at the level of the optic chiasm (white arrow) in a 9-month-old NF1 minipig. **b** Axial T1-weighted MRI of a normal optic chiasm from a 16-month-old NF1 minipig. **c** H&E staining of the optic pathway lesion from an NF1 minipig shows hypercellularity. **d** H&E staining of an optic nerve from an NF1 minipig. **e** H&E staining of an optic nerve from a wild-type minipig. **f** Iba1 immunohistochemistry of the optic pathway lesion from an NF1 minipig shows increased microglial infiltration. **g** Iba1 immunohistochemistry of an optic nerve from an NF1 minipig. **h** Iba1 immunohistochemistry of an optic nerve from a wild-type minipig. **i** Ki67 staining of an optic pathway lesion from an NF1 minipig shows a low proliferative index. **j** Ki67 staining of an optic nerve from an NF1 minipig. **k** Ki67 staining of an optic nerve from a wild-type minipig. Scale bars, 50 µM

OPGs occur in 15–20% of children with NF1, where >80% are located within the optic nerves and chiasm^1,6,40–42^. These slow-growing benign tumors are typically identified by magnetic resonance imaging (MRI), often revealing focal enlargement of the optic nerve and/or chiasm^42–44^. One of seven NF1 minipigs imaged by MRI for OPG development exhibited a mass involving the optic chiasm that extended ventrally into the surrounding tissue (Fig. 3a, b, Table 1). Histopathology of this lesion revealed hypercellularity, microglial infiltration, and a low Ki67 proliferative index, similar to OPGs reported in NF1 patients and genetically engineered mouse models (Fig. 3c–k)^21,42,44–47^.

### Cells from NF1 minipigs undergo loss of heterozygosity

**Fig. 4:**
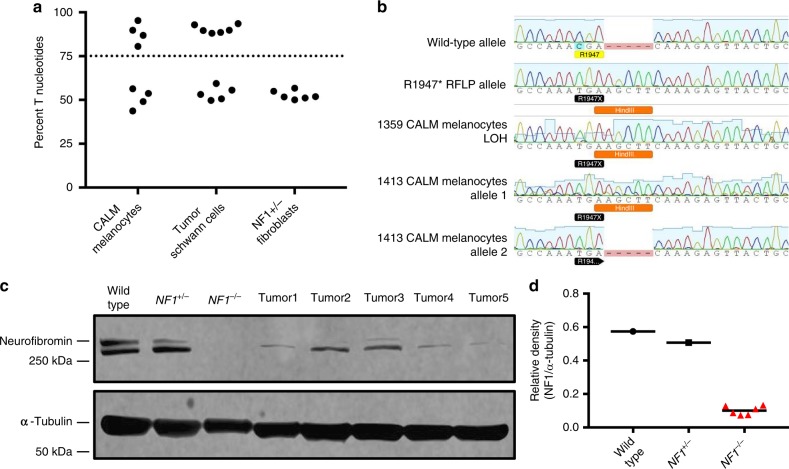
A subset of CALM-derived melanocytes and neurofibroma-derived Schwann cells show loss of heterozygosity. **a** The chromatogram trace peak area of T nucleotides at the level of the R1947* point mutation (CGA (R)→TGA (*)) relative to total trace peak area is displayed as the percentage of T nucleotides. Samples with >75% percent T nucleotides were considered to have undergone LOH. Samples with less than 75% T nucleotides were considered to have retained the wild-type allele. 4/8 melanocyte cultures derived from CALMs and 6/11 Schwann cell cultures from neurofibromas show LOH. In contrast, none of the six fibroblast cultures isolated from normal NF1 minipig skin (*NF1*^*+/*−^ fibroblasts) show LOH. **b** Sanger sequencing shows various mechanisms of LOH. An example chromatograph of a wild-type allele and R1947* RFLP allele isolated by TOPO cloning and sequenced are shown at the top. Melanocytes isolated from a CALM from NF1 minipig 1359 show complete conversion to the RFLP allele as seen by PCR population allele sequencing. Melanocytes isolated from a CALM from NF1 minipig 1413 show LOH with incorporation of the *NF1*^R1947*^ mutation, but not the *Hin*dIII RFLP site, as seen by TOPO cloning. **c** Five Schwann cell lines from NF1 minipig neurofibromas (tumors 1–5) with LOH show loss of neurofibromin protein expression. This western blot was cropped to improve the conciseness of the presentation. The full-length blot is presented in Supplementary Figure 7. This western blot is representative of three experiments. **d** Relative density quantification of neurofibromin protein expression to α-tubulin. Wild-type Schwann cells from wild-type minipig sciatic nerve, *NF1*^*+/*−^ Schwann cells from NF1 minipig sciatic nerve, *NF1*^−/−^ immortalized human *NF1*^−/−^ Schwann cell line

Bi-allelic inactivation of the *NF1* gene through a “second-hit” mutation in a subset of Schwann cells has been demonstrated in neurofibromas from human patients^48–51^. This phenomenon has also been described in melanocytes isolated from CALMs^50,52^. To evaluate this in our minipig model, CALM-derived melanocytes and neurofibroma-derived Schwann cells were subjected to DNA sequencing. LOH at the *NF1* locus was detected in a subset of CALMs and neurofibromas, by either a gene conversion event or large deletion (Fig. 4a). Interestingly, one CALM showed conversion to R1947* without RFLP incorporation, suggesting partial gene conversion or a point mutation in the wild-type allele (Fig. 4b). Schwann cells were subjected to western blot analysis for neurofibromin protein expression, which confirmed the DNA sequencing results in Schwann cells isolated from tumors (Fig. 4c, d). To validate the biochemical defect in Ras signaling associated with *NF1* loss, Schwann cells from neurofibromas or sciatic nerves were starved overnight, serum stimulated, and analyzed by western blot for active Ras (Ras-GTP). Variable levels of Ras activation were seen in Schwann cells isolated from tumors with LOH compared to wild-type or *NF1*^*+/−*^ Schwann cells, despite >90% purity in Schwann cell cultures (Supplementary Figure 3).

### NF1 minipigs display other NF1-associated phenotypes

Electron microscopy (EM) of NF1 minipig optic nerves showed myelin decompaction, a phenotype observed in mouse models and correlated with white matter enlargement and behavioral deficits in NF1 patients^53,54^ (Supplementary Figure 4). Lesions resembling iris hamartomas, or Lisch nodules, were noted in two of the three NF1 minipigs examined by slit-lamp (Table 1, Supplementary Figure 5). Tibial diaphyseal narrowing was observed in 15.4% (2/13) of NF1 minipigs that underwent X-ray analysis (Table 1, Supplementary Figure 5). Blood pressure, weight, height, length, and head circumference were measured periodically in a cohort of NF1 minipigs and their wild-type littermate controls (Supplementary Figure 6). At 8 months of age, NF1 minipigs weighed significantly less than their wild-type siblings. All 12 NF1 minipigs (five F0 animals and seven F1 animals) that underwent MRI analysis also underwent full-body computerized tomography (CT) scanning and no other tumors were observed (Table 1).

### NF1 minipigs as a preclinical model for pharmacology studies

**Fig. 5:**
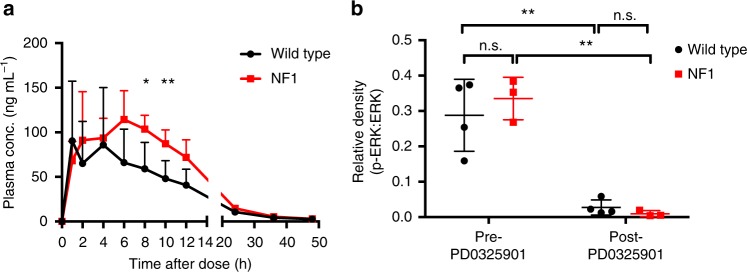
The NF1 minipig as a preclinical pharmacology model. **a** Plasma concentration-time plot of PD0325901 in wild-type (*n* = 4) and NF1 (*n* = 4) minipigs following a single dose of PD0325901 administered orally at 0.79 mg kg^−1^. Error bars represent standard deviation of the mean. **b** Relative levels of p-ERK in isolated PBMCs from wild-type and NF1 minipigs are shown. Whole blood was collected before (pre-PD0325901) and 4 h after (post-PD0325901) drug administration, then stimulated with PMA ex vivo to induce MAPK pathway activation in PBMCs. PD0325901 suppressed ERK phosphorylation by >90% in PBMCs within 4 h in both wild-type (*n* = 4) and NF1 (*n* = 3) minipigs. Lines represent mean with standard deviation. Statistical analysis: n.s.—not significant, *P = 0.03, **P = 0.01, paired *t*-test. The full-length blot is presented in Supplementary Figure 8

It is useful to have a large animal disease model for testing safety and efficacy of novel pharmaceutical compounds prior to human studies. To demonstrate that NF1 minipigs represent a relevant preclinical model, we performed pharmacokinetic and pharmacodynamic analysis of PD0325901, a MEK inhibitor currently in clinical trials for NF1-related tumors (clinicaltrials.gov). A single dose of 0.79 mg kg^−1^ PD0325901 was administered orally to four wild-type and four NF1 littermates and plasma was collected over time for pharmacokinetic analysis. PD0325901 was detectable in the plasma within 1 hour of administration, and the mean maximum plasma concentration of PD0325901 was 125 ± 47 ng mL^−1^, a value higher than the reported PD0325901 plasma concentration (99 ng mL^−1^) required for a pharmacodynamic effect in other preclinical models (Fig. 5a)^55^. Notably, NF1 minipigs showed significantly higher plasma levels of PD0325901 than their wild-type littermate controls at 8 h (wild-type 59 ± 30 ng mL^−1^ vs. NF1 104 ± 15 ng mL^−1^) and 10 h (wild-type 48 ± 20 ng mL^−1^ vs. NF1 87 ± 15 ng mL^−1^) after drug administration, though estimated pharmacokinetic parameters showed no statistically significant differences between wild-type and NF1 minipigs (Fig. 5a, Supplementary Table 3). To measure the pharmacodynamic effect of PD0325901 in minipigs, peripheral blood mononuclear cells (PBMCs) were evaluated for suppression of phorbol-myristate acid (PMA)-stimulated ERK phosphorylation, a pharmacodynamic biomarker of MEK inhibitors^56,57^. A single dose of PD0325901 was sufficient to suppress ERK phosphorylation in PBMCs by 98.1% (±0.7) in NF1 minipigs and 95.8% (±1.2) in wild-type minipigs (Fig. 5b). These results suggest that NF1 minipigs could serve as a valuable preclinical model for pharmacokinetic and pharmacodynamic analysis of targeted therapies.

## Discussion

We describe here, the first minipig model of NF1 that exhibits the spectrum of clinical features seen in patients. NF1 minipigs meet NIH diagnostic criteria for NF1, and a subset of minipigs develop neurofibromas and OPG. This is also the first animal model of NF1 to exhibit CALMs, one of the most common clinical manifestations in humans. Both melanocytes and Schwann cells from NF1 minipigs undergo spontaneous LOH, a critical step in disease progression. To our knowledge, spontaneous LOH in the cells of origin of neurofibroma and CALM has not been demonstrated in any other animal model of NF1. Interestingly, porcine Schwann cells with LOH show variable evidence of hyperactive Ras signaling in vitro. This may be indicative of a more complex biochemical process associated with *NF1* loss in neurofibromas, such as activation of dual specificity phosphatases^39^.

Similar to the human condition, our model shows variability in expression of NF1 phenotypes. For instance, NF1 minipig CALMs vary greatly in size and number between animals. Additionally, only a subset of minipigs develop OPG and neurofibromas. A clear strength of our system is that the NF1 minipig closely mimics the age of onset for NF1-related phenotypes in humans. In humans, CALMs are often present at birth, and neurofibromas tend to develop around puberty. CALMS were observed perinatally in all pigs, and minipig neurofibromas develop at around 4 months of age (puberty ~5–6 months). While OPG is typically diagnosed at a young age in humans, this phenotype was not noted in the NF1 minipig until 9 months of age (adulthood). However, it is likely that OPG was present at a young age and could not be diagnosed until MRI was performed, as these tumors are often asymptomatic.

While various model systems have been implemented to induce tumor development in mice, these have not proven to be representative of the complex natural history of NF1-associated tumorigenesis^20,21^. For example, *Nf1*^*+/*−^ mice do not develop peripheral nerve sheath tumors or other hallmarks of NF1 syndrome (Table 2)^22,23^. Interestingly, a subset will develop less common NF1-associated tumors later in life, including neurofibrosarcoma, pheochromocytoma, and myeloid leukemia^22,23^. It is possible that we would see these manifestations in the NF1 minipig, but Ossabaw minipigs have a lifespan of up to 15 years in captivity and were only monitored for 16 months for the purposes of this study. In comparison to the analogous mouse model described by Jacks et al.^22^ and Brannan et al.^23^, our NF1 minipig model manifests the more common hallmarks of the disease (Table 2). In fact, the original *Nf1*^*+/*−^ mouse models share no phenotypes with our *NF1*^*+/*−^ minipig model (Table 1).

The genetic, anatomic, and physiologic similarities between minipigs and humans make them an optimal model for studying the biology and natural history of NF1. The minipig provides a human-sized platform for imaging studies to better evaluate tumor natural history and therapeutic efficacy and to develop methods for early detection of NF1-associated tumors. Their large size also makes it feasible to perform longitudinal blood and tissue sampling for identification of diagnostic biomarkers and evaluation of NF1-targeted therapies. The NF1 minipig may prove particularly valuable for studying long-term toxicity of targeted therapies in both pediatric and adult populations, as many druggable targets show significant homology between pig and human. Further, this model provides the opportunity to evaluate drug penetrance through the blood–nerve and blood–brain barriers. These studies will be critical in evaluating efficacy of treating NF1-related tumors including neurofibromas and OPGs.

Thus far, other important NF1-associated tumors including plexiform neurofibromas, malignant peripheral nerve sheath tumors, and gastrointestinal stromal tumors have not been observed in our model. We will continue monitoring NF1 minipigs as they age for the development of these and other clinical manifestations. To model more aggressive phenotypes, the NF1 minipig could be augmented with additional germ line or somatic mutations. For example, *Tumor Protein p53 (TP53)*, a tumor suppressor gene that is often found co-mutated with *NF1* in malignant peripheral nerve sheath tumors, is in close proximity to the *NF1* gene (~9 MB) in the swine genome. This allows for the development of a minipig in which both *NF1* and *TP53* are mutated in cis, similar to previously described mouse models of malignant peripheral nerve sheath tumors^58,59^. Swine also harbor the * Polycomb Repressive Complex 2 Subunit* *(SUZ12)* gene, which is recurrently inactivated in NF1-associated malignancies and could be mutated or deleted with *NF1* to drive the formation of additional tumor types. Future studies will also evaluate NF1-associated cognitive deficits and neuro-developmental disabilities in our minipigs^60,61^. We predict that our NF1 minipig model will ultimately accelerate treatment options for patients and serve as a platform for studying the natural history of this intractable disease.

**Table 2:** Comparison of mouse, minipig, and human NF1-associated phenotypes^1,4,5,22,23,80^

NF1 animal model	Age at puberty	Lifespan	Average age of onset						
			OPG	Cutaneous neurofibroma	Lisch nodule	CALM	Pheochromocytoma	Myeloid leukemia	Tibial diaphyseal narrowing
Mouse (*Mus musculus*)	6–8 weeks	1–3 years	n/a	n/a	n/a	n/a	15–28 months	17–27 months	n/a
Ossabaw minipig (*Sus scrofa*)	5–6 months	15 years	9 months	4 months	Pre-puberty	Birth	n/a	n/a	Pre-puberty
Human	10–15 years	70–80 years	3–5 years	10–15 years	Pre-puberty	Birth	39 years	2 years	6–12 months

## Methods

### TALEN design, assembly, and RNA synthesis

All TALENs were designed using the TALE-NT software and assembles using standard methods^62^. Linearized TALEN DNA was transcribed in vitro using the mMessage Machine T3 kit (Ambion). Synthesis reactions were assembled in a 20 µL reaction with 1 µg linearized plasmid DNA, 1× NTP/CAPs (Ambion), 1× reaction buffer (Ambion), and 2 µL enzyme mix (Ambion). Reactions were incubated for 2 h at 37 °C, treated with Turbo DNase (Invitrogen), then cleaned up with the RNeasy Mini Kit (Qiagen).

### Cell culture and transfection of swine embryonic fibroblasts

Ossabaw minipig fibroblasts isolated from day 30–36 embryos were cultured in 1× high-glucose DMEM (Invitrogen) with 10% FBS (Atlas Biologicals), 2 mM l-glutamine (Corning), 10 mM HEPES buffer (Lonza), 1× penicillin/streptomycin solution (Corning), 5 µg mL^−1^ Apo-Transferrin (Sigma), 20 ng mL^−1^ recombinant human IGF-1 (R&D Systems), and 25 ng mL^−1^ recombinant human EGF (R&D Systems) and transfected using the Neon Transfection System (Thermo Fisher Scientific). Briefly, each transfection reaction included 600,000 fibroblasts, 0.5 μg of RNA from each transcribed TALEN, and 0.2 nmol HDR oligonucleotide, and the transfection reaction was pulsed once at 1800 V for 20 ms using the Neon™ transfection system (Thermo Fisher Scientific). Transfected cells were cultured 3 days at 30 °C, before splitting for RFLP analysis and plating for colony isolation at 38.5 °C. Individual colonies were collected in 10-cm dishes, where 80–250 transfected cells were plated and allowed to grow for 10–14 days and individual colonies were aspirated under gentle trypsinization.

### Detection and sequence validation of gene modification

Transfected cells harvested at day three were prepared for PCR by pelleting and resuspending in PCR-safe lysis buffer (10 mm Tris·Cl, pH 8.0; 2 mM EDTA; 2.5% (vol/vol) Tween-20; 2.5% (vol/vol) Triton X-100; 100 μg mL^−1^ proteinase K) at ∼1000 cells per μL, followed by incubation at 50 °C for 60 min and 95 °C for 15 min. Typically, 1 μL of prepared lysate was used in a 2× AccuStart II PCR SuperMix (QuantaBio); all other applications were according to the manufacturer’s protocol. Gene modification in individual colonies was detected by RFLP analysis and direct sequencing of PCR amplicons, characterized by TOPO cloning (Invitrogen) and sequencing.

### Animal husbandry and cloning

NF1 minipigs were produced under license of chromatin transfer technology from Hematech to Cooperative Resources International Center for Biotechnology (CRI_ICB), Verona, WI)^63^. All animal work was performed in Recombinetics facilities under its Animal Welfare Assurance #A4728-01 and the University of Minnesota under its Animal Welfare Assurance #A3456-01. All animal protocols were reviewed and approved by the Institutional Animal Care and Use Committee (IACUC).

### H&E, toluidine blue, and immunohistochemical staining

H&E staining was performed on 3.5-µM-thick sections prepared from paraffin blocks of formalin-fixed tissues. For mast cell staining, a toluidine blue stock solution (10×) was prepared by diluting 1 g of toluidine blue O (Fisher Chemical, T161) in 100 mL of ethanol 70%. The stock solution was freshly diluted in sodium chloride 1% with pH adjusted to 2.0–2.5 with glacial acetic acid. Sections were immersed in toluidine blue solution for 3 min, rinsed three times in distilled water, then quickly dehydrated and cleared in Clear-Rite (Thermo Scientific) before mounting. For immunohistochemistry, heat-induced epitope retrieval (HIER) was performed with target retrieval solution pH9 (Dako, S2367) except for S100β staining where no HIER was used. A standard protocol was used with primary antibodies incubated 1 h at room temperature (RT): c-Kit (1:200, Cell Signaling Technology, #3074), GFAP (1:3000, Dako, Z0334), Ki-67 (1:1000, BD Pharmingen, 556003), S100β (1:10000, Dako, Z0311). Biotinylated secondary anti-rabbit (1:2000 or 1:50,000), anti-mouse (1:5000 Vector Laboratories, Burlingame, CA) IgG antibodies were incubated 30 min at RT. Pictures were taken with an Axio Imager M1 microscope and Axiovision software (Zeiss).

### Minipig sedation, radiographic imaging, and euthanasia

Pigs were transported to the University of Minnesota Veterinary Medical Center (VMC) for housing and radiographic imaging. Minipigs were housed in stalls or kennels sized per the Guide for the Care and Use of Laboratory Animals. Minipigs were housed individually, fed a standard pig diet, and allowed water. Minipigs were fasted for 12–18 h prior to anesthesia.

Anesthesia was induced with telazol (2.2–4.4 mg kg^−1^), xylazine (2 mg kg^−1^), ±ketamine (20 mg kg^−1^) intramuscularly (IM), then the animals were intubated with balloon-cuffed 7.5–10 mm endotracheal tubes. Anesthesia was maintained with 1–5% isoflurane. Minipigs were mechanically ventilated at 100% oxygen with a volume of 340–1000 mL (depending upon size) and pressure 15–22 cm water, to maintain an end tidal CO_2_ of 35–45 mmHg. An 18–22-gauge IV catheter was placed in a peripheral ear vein or cephalic vein for administration of IV fluids and euthanasia solution. IV fluids (Lactated Ringers Solution or 0.9% saline) were given to minipigs that had multiple imaging procedures. Pulse, respirations, blood oxygen saturation, CO_2_ level, and blood pressure were monitored continuously and recorded every 5–15 min.

Once anesthetized, minipigs were transported to the desired imaging area: X-ray, CT, or MRI. Typically, minipigs receiving all three modalities would start in CT, move to MRI, then to X-ray. The bore of the MRI limited the size of minipigs, which could be scanned. Therefore, minipigs over 90 kg were imaged by CT, then X-ray, then MRI of the head only post-mortem.

*CT acquisition*: minipigs were placed in the supine position and a single, non-contrast enhanced dataset of the chest–abdomen–pelvis region was acquired using a Toshiba Aquilion-64 CT scanner (Toshiba Medical Systems, software version V3.35ER006). Images were acquired in a helical mode using the full 64-row detector at an image thickness of 0.5 mm, with a standard technique of 120 kV, 200 mAs, 0.5 s rotation time, pitch factor of 0.828, convolution kernel FC03. The raw image dataset was reconstructed into 2.0 mm thick by 2.00 mm image interval datasets in three separate convolution kernels to accentuate soft tissue (FC03), bone (FC30), and lung parenchyma (FC52).

*MRI acquisition*: MRI data were obtained on a GE Signa HDxT 3.0T MRI system (GE Medical Systems, software version HD 16.0_V03_181638.a). Brain sequences: The minipig was placed in supine position and imaged using a cervical–thoracic–lumbar (CTL)-phased array coil. An initial set of T2-weighted images of the skull in the sagittal, coronal, and transverse planes (TR: 3000–7000 ms; TE: 123–125 ms; echo train length: 1; flip angle: 90°; resolution: 5.0 mm slice thickness × 1.8 mm) were acquired. This was followed by a T2 Fluid Attenuation Inversion Recovery (FLAIR) dataset in the coronal plane (TR: 8000 ms; TE: 100–102 ms; echo train length: 24; flip angle: 90°; inversion time: 2250 ms; resolution: 5.00 mm slice thickness × 1.8 mm spacing), a T1-weighted dataset of the same area (TR: 600–1000 ms; TE: 123–125 ms; echo train length: 1; flip angle: 90°; resolution: 5.0 mm slice thickness × 1.8 mm spacing), and an oblique-coronal T2 with fat saturation dataset, angled to image approximately parallel with the optic nerve, (TR: 8000 ms; TE: 100–102 ms; echo train length: 24; flip angle: 90°; inversion time: 2250 ms, resolution: 3.0 mm slice thickness × 0.3 mm spacing). An oblique-coronal T1 dataset of the same images was also acquired (TR: 600–1000 ms; TE: set to min full; echo train length: 3; resolution: 3.0 mm slice thickness × 0.3 mm spacing). In some minipigs, a 3D T1 (fast spoiled gradient echo) sequence was obtained (TR: variable; TE: set to min full; prep time: 450 ms; flip angle: 12; resolution: 1.0 mm × 1.0 mm × 1.2 mm slice thickness). Spine sequences: short-tau inversion recovery images of the cervical, thoracic, and lumbar spine were acquired in the sagittal plane (TR: 3000–7000 ms; TE: 25–55 ms; echo train length: 10–12; flip angle: 90°; inversion time: 190 ms; resolution: 3.0 mm slice thickness × 0.3 mm spacing). Transverse T2 images with fat saturation were acquired (TR: 8000 ms; TE: 100–102 ms; echo train length: 24; flip angle: 90°; inversion time: 2250 ms, resolution: 5.0 mm slice thickness × 3.0 mm spacing). Transverse T1-weighted images were acquired (TR: 600–1000 ms; TE: 123–125 ms; echo train length: 1; flip angle: 90°; resolution: 5.0 mm slice thickness × 3.0 mm spacing). Dorsal 3D T2 with fat saturation images were acquired (TR: 2500 ms; TE: 101 ms; echo train length: 100; flip angle: 90°; resolution: 1.0 mm × 1.0 mm × 1.0 mm).

Radiographs of minipigs under 40 kg were imaged using the VMC Small Animal Digital X-ray (Sedecal Vet-X Technology) digital radiograph system: RAD positional, high frequency generator, Toshiba Rotanode tube E7-23X, software Vieworks Co. Ltd. VXvue version 1.0.0.82b10, DR detector: ViviX 17 × 17 digital panel, amorphous silicon sensor (a-SI) high speed Gadox scintillator, 140-μm pixel pitch, 9.2 million pixels (3008 × 3072), 4096 gray scale (14 bits A/D). Images taken were of the entire spine, cervical to sacrum, lateral and ventrodorsal (VD), and both hind legs (tibia or tibia/fibula with two views (VD and lateral)). Imaging parameters were stored as a part of the DICOM information with each image.

Radiographs of minipigs over 40 kg were imaged using the VMC Large Animal X-ray: (Varian Medical Systems Tube (2016, type RAD-44, housing mode “Sapphire”); Acoma tower, Shimadzu control unit). Images taken were of the entire spine, cervical to sacrum, lateral and VD, and both hind legs (tibia or tibia/fibula with 2 views (VD and lateral)). Imaging parameters used were based upon size and resulting quality, with a range of 84–110 peak kilovoltage (KVP), 500–630 mA, and 100–250 mAs. X-ray images were captured on cassette and read into PACS (Picture Archiving and Communication System).

Following imaging, the minipigs were transported to the Veterinary Diagnostic Laboratory (VDL) for euthanasia and necropsy. Prior to transport, an IM dose of telazol (2.2 mg kg^−1^), xylazine (1 mg kg^−1^), and ±ketamine (10 mg kg^−1^) was given.

### Optic pathway immunohistochemistry and pathological analysis

Formalin-fixed paraffin-embedded sections were subjected to immunohistochemistry using cell-type-specific antibodies^64^. The optic nerves and chiasms were dissected from euthanized animals and fixed in formalin prior to paraffin embedding and sectioning. Five-micrometer sections were deparaffinized, treated for citrate antigen retrieval, and incubated in 5% serum blocking solution for 1 h at RT. Slides were next incubated with Iba1 (1:1000 dilution; WAKO; 019–19741) or Ki67 (1:1000 dilution; Abcam; ab15580) primary antibodies overnight at 4 °C, followed by a 1-h exposure to biotinylated species-specific secondary antibodies (Vector Laboratories) and development using the Vectastain Elite ABC kit (Vector Laboratories) according to the manufacturer’s instructions. H&E staining was accomplished using standard methods. Images were acquired on an Olympus BX51 microscope using cellSens Entry imaging software.

### Isolation and culture of primary minipig cell lines

Minipig skin fibroblasts were isolated from ear clips transported in 1× HBSS (Corning) with 1× antibiotic/antimycotic (AA; Corning). Hair and subcutaneous fat were trimmed from specimens, rinsed in 1× phosphate-buffered saline (PBS, Invitrogen) with 1× AA, then lathered with povidone-iodine (Betadine). Specimens were transferred into a series of tubes beginning with 70% ethanol and three sets of 1× PBS with 1× AA washes. Underlying hypodermis was trimmed and discarded using sterile forceps and scalpel. Tissues were minced and transferred to a conical tube containing full growth media (1× high-glucose DMEM (Invitrogen) supplemented with 10% FBS (Atlas Biologicals), 2 mM l-glutamine (Corning), 10 mM HEPES buffer (Lonza), 1× penicillin/streptomycin solution (Corning), 5 μg mL^−1^ Apo-Tranferrin (Sigma), 20 ng mL^−1^ recombinant human IGF-1 (R&D Systems), and 25 ng mL^−1^ recombinant human EGF (R&D Systems)), 200 U mL^−1^ collagenase, and 1× AA and incubated at 37 °C on a tube rotator for 16 h. Fibroblasts were centrifuged at 300×*g* for 10 min followed by two washes in 1× PBS and centrifugation repeated. Cells were resuspended in full growth media containing 1× AA, counted with a hemocytometer, and plated at 2.8 × 10^4^ cells per cm^2^ with media changes every 3 days.

Isolation of porcine Schwann cells was adapted from a protocol for human Schwann cells^65^. Porcine sciatic nerves (1–2 inches) were removed upon euthanasia and placed immediately in ice-cold DMEM with antibiotic–antimycotic (GIBCO), then transported to the laboratory within 2 h. Nerves were cut into 1 cm^3^ pieces and incubated at 37 °C, 5% CO_2_ in pre-treatment medium (PM) containing DMEM with high glucose (GIBCO) supplemented with 10% fetal bovine serum (FBS) (GIBCO), gentamicin (50 mg mL^−1^) (GIBCO), fungizone (2.5 mg mL^−1^) (GIBCO), forskolin (2 μM) (Calbiochem), and recombinant neuregulin-b1 (NRG1) (10 ng mL^−1^) (R&D Systems). Pre-treatment medium was replaced every 2 days. After 7 days, nerves were dissociated for 3–5 h in DMEM containing 10% FBS, collagenase type I (130 U mL^−1^) (GIBCO), dispase II (2.5 mg mL^−1^) (Roche), gentamicin (50 mg mL^−1^), fungizone (2.5 mg mL^−1^), penicillin–streptomycin (GIBCO), at 37 °C. Nerves were then mechanically dissociated by trituration using a 5 mL pipet, then centrifuged for 10 min at 1000 r.p.m., resuspended in PM, and incubated at 37 °C, 5% CO_2_ on poly-l-lysine and laminin-coated plates. To coat plates, poly-l-lysine (0.05 mg mL^−1^) (Sigma) was applied for 30 min at RT, then removed and then plate washed three times with PBS. Mouse laminin (10 mg mL^−1^) (GIBCO) was applied for 1 h at RT, then removed and replaced with DMEM and 10% FBS for 30 min at RT. Plates were kept in PBS until use. Media was changed every 2–3 days and cells were passaged when they reached 90% confluence and seeded in fresh coated plates at 30,000 cells per mL. Geneticin (G418) (Corning, 100 μg mL^−1^) was added to cultures for 48 h to eliminate contaminating fibroblasts^66^. G418 treatment was performed up to two times with a 1-week rest period between treatments. Schwann cells were cryopreserved in DMEM with 20% FBS and 10% dimethyl-sulfoxide (DMSO) (Sigma). Schwann cells were isolated from tumors as above, with minor modifications. Epidermal tissue was discarded and tumor tissue (4 mm–2 cm) was cut into ~5 mm^3^ pieces and incubated for 7 days in PM, then incubated overnight in dissociation media. Tumor-associated fibroblasts were prominent and relatively resistant to G418 treatment and were therefore removed by differential trypsinization, colony picking, serum-free media, or magnetic cell separation according to the manufacturers protocol using an anti-NGFR antibody and positive selection (eBioscience 50-112-2782)^65^. For differential trypsinization, cells were washed with PBS and then incubated for 1–2 min in 0.05% trypsin diluted in PBS (Gibco). The supernatant was removed, and remaining cells were harvested with 0.25% trypsin. These cells were counted and re-seeded onto fresh coated plates.

Isolation and growth of porcine melanocytes was adapted from the human protocol^67^. Skin biopsies of 1 cm^3^ were incubated overnight in 10 mg mL^−1^ dispase II (Boehringer Mannheim) to separate epidermis from dermis. Epicutaneous sheets were carefully removed with forceps and dissociated for 10 min at 37 °C in 500 μg mL^−1^ trypsin/EDTA. Melanocytes were cultured in media containing DMEM high glucose supplemented with 10% FBS, 10 ng mL^−1^ porcine stem cell factor (Kingfisher), 100 pM cholera toxin (Sigma), 200 nM 12-O-tetradecanoylphorbol-13-acetate (TPA) (CST), and 10 nM endothelin-1 (BACHEM). Media was changed every 2–3 days and fibroblasts were removed using G418, as for Schwann cell cultures. Melanocytes were identified by morphology and pigmentation and were considered pure when there was <5% fibroblast/keratinocyte contamination. While there was often significant contamination in the first few days after dissociation, most cultures were >99% pure after the first passage, as keratinocytes do not survive under these growth conditions and fibroblasts were successfully eliminated with G418 treatment.

### NF1 LOH analysis

To measure LOH at the *NF1* locus in tumor-derived Schwann cell and CALM-derived melanocyte cultures, the trace peak area at the location of the point mutation (C → T) was measured in each DNA sequencing chromatogram, and the percent area of each peak compared to total area was calculated using the following packages in R: plyr, magrittr, tidyverse, sangerseqR^68–72^. In heterozygous cells, the percent of T alleles should be about 50% and was found to be between 50% and 57% in fibroblasts from normal NF1 minipig skin. In cells that have lost the wild-type allele, the percentage of T nucleotides should be about 100%. DNA isolated from tumor-derived Schwann cells and CALM-derived melanocytes ranged from 44 to 95%, with a distinct gap between 64 and 80%. The cutoff for LOH was therefore set at 75% T nucleotides to account for contamination with *NF1*^*+/*−^ cells and inherent limitations of the sequencing software^73^.

### Western blotting

Cells were harvested in trypsin, washed once with 1× PBS, snap-frozen in liquid nitrogen, and stored at −80 °C as dry pellets until analysis. Cells were lysed in RIPA buffer (Sigma) containing phosphatase and protease inhibitors (Sigma) and centrifuged for 15 min at 4 °C at 15,000 r.p.m. Supernatants were used for analysis. Total protein concentration was estimated by BCA assay (Pierce). Twenty micrograms of total protein was resolved on a 4–12% Nupage Bis-Tris gel (Thermo) and transferred overnight at 4 °C to an immun-blot PVDF membrane (Bio-Rad) using an XCell II Blot module (Thermo). Blots were blocked for 1 h at RT in PBS supplemented with 0.1% Tween-20 (Sigma) and 5% non-fat dry milk (Bio-Rad). Blots were cut into strips and incubated with primary antibody overnight at 4 °C with rotation. Blots were then washed three times for 5 min and incubated with secondary antibody for 1 h at RT. Blots were washed as described and developed with WesternBright Quantum reagents (Advansta). Bands were visualized using a Li-Cor Odyssey Fc Imaging System. Semi-quantitative levels of each band were analyzed by densitometry using Li-Cor Image Studio software, and the relative values normalized to α-tubulin are indicated numerically under each lane. Antibodies used for this analysis were anti-neurofibromin (SCBT sc376886)^74^ or anti-α-tubulin (CST 2144)^75^, anti-mouse IgG HRP (Pierce 31431), and anti-rabbit IgG-HRP (SCBT sc2313).

### PD0325901 formulation and dosing

PD0325901 (Pfizer) was obtained from SelleckChem (Houston, TX) and underwent testing at the University of Minnesota’s Institute for Drug Discovery for confirmation of molecular identity and analysis of purity using an acetonitrile/water/formic acid liquid chromatography (LC) conditions. PD0325901 was determined to be >99% pure by LC/mass spectrometry (MS) and nuclear magnetic resonance spectroscopy (NMR) analysis. PD0325901 was formulated for oral administration in aqueous 0.5% (w v^−1^) methylcellulose solution with 0.2% (v/v) polysorbate 80 (Tween 80) to a concentration of 4 mg mL^−1^ and sonicated to form a suspension. A single oral dose was administered to four wild-type and four NF1 minipigs at 0.79 mg kg^−1^. The required volume of drug for each animal was based on individual body weight determined on the day of administration.

### Pharmacokinetic analysis of PD0325901

Whole blood was collected from the jugular vein into sodium heparin vacutainer tubes prior to PD0325901 administration (pre), and 1, 2, 4, 6, 8, 10, 12, 24, 36, and 48 h after PD0325901 administration. Samples (1.5 mL) were immediately transferred to a 1.7 mL microfuge tube and centrifuged at 2000×*g* for 10 min in a tabletop refrigerated centrifuge at 4 °C. Plasma was subsequently transferred to a cryovial and stored at −80 °C until analysis.

Following the addition of an internal standard (30 ng of PLX4720), plasma samples (0.15 mL) were extracted with 1.25 mL of ethyl acetate using a multi-tube vortex for 10 min. Following centrifugation at 15,000×*g* for 5 min, the supernatant was removed and evaporated to dryness using a nitrogen evaporator (Zymark Turbo Vap LV, Hopkinton, MA) set at 37 °C. The residue was reconstituted with 125 µL of mobile phase^76^.

Detection and quantification of PD0325901 was performed using a high-performance liquid chromatograph (Agilent 1100 Series, Santa Clara, CA) coupled with an API 4000 triple quadrupole instrument (MDS-SCIEX, Concord, Ontario, Canada). The chromatographic separation was performed with a ACQUITY UPLC BEH C18 column, 50 mm × 2.1 mm, 1.8 µm (Milford, MA) with a mobile phase containing (40:60) DI water with 0.1% formic acid: acetonitrile with 0.1% formic acid, at a flow rate of 250 µL min^−1^, with the column temperature set at 30 °C. Mass spectrometric detection was performed using MRM (multiple reaction monitoring) in negative ionization mode. Source conditions were as follows: the turbo-gas temperature was set at 400 °C, and the ion spray needle voltage was optimized at −4500 V. The mass spectrometer was operated at unit resolution for both Q1 and Q3 in the MRM mode, with a dwell time of 100 ms per MRM channel. The precursor/product ion pairs monitored were *m/z* 481–>398 for PD0325901 and *m/z* 412–>305 for the internal standard (IS) (PLX4720). Ion source gases 1 and 2 were set at 20 and 50 (arbitrary units), respectively; the curtain gas was at 50 (arbitrary units) and the collision gas at 4 (arbitrary units). The collision energy was set at −22 eV for PD0325901 and −38 eV for the IS. Data acquisition was performed with analyst 1.4.1 software (MDS-SCIEX, Concord, Ontario, Canada).

PD0325901 was obtained from MilliporeSigma (St. Louis, MO) and the internal standard was obtained from Cayman Chemicals (Ann Arbor, Michigan). Minipig plasma for calibrators and standards were obtained from Valley Biomedical (Winchester, VA). The assay was linear in the range of 1–5000 ng mL^−1^, using 1/X weighting. Method validation accuracy was 100.5% and the total variability was 6.8% (6.4% within day and 2.1% between days).

PD0325901 plasma concentration-time data from oral administration were analyzed using noncompartmental methods as implemented in R (version 3.4.1) R Studio PKNCA package (version 0.8.1)^77^. The pharmacokinetic parameters included area under the concentration-time curve from time 0 to 48 h (linear up, log down), maximum concentration (Cmax), time to Cmax (Tmax), and half-life (*t*_1/2_).

### Pharmacodynamic analysis of PD0325901

Blood samples were collected immediately before PD0325901 administration and 4 h after PD0325901 administration. Whole blood was treated ex vivo with 200 nM 12-*O*-tetradecanoylphorbol-13-acetate or PBS for 10 min at 37 °C within 1 h of being drawn. Peripheral  blood mononuclear cells (PBMCs) were isolated, washed, and dry pellets were snap-frozen and stored at −80 °C. Cells were lysed as described above. Relative quantification of p-ERK to total ERK was completed using an automatic Simple Western apparatus, Wes (Protein Simple), following the manufacturer’s protocol. Primary antibodies used for this analysis were rabbit anti-p-ERK (CST #4695S)^78^ and rabbit anti-ERK (CST #4370S)^78^.

### Ras activation assay

Schwann cells were serum starved with DMEM supplemented with 0–1% FBS overnight before stimulation with complete media. Affinity precipitation of Ras-GTP was performed using the active Ras pull-down and detection Kit (Thermo Scientific), following the manufacturer’s protocol. Twenty microliters of lysate was removed prior to affinity precipitation for quantification of total Ras. A BCA assay was not performed due to low total protein. Relative quantification of Ras-GTP to total Ras was done using the provided pan Ras antibody on an automatic Simple Western apparatus, Wes (Protein Simple), following the manufacturer’s protocol.

### Immunofluorescence microscopy and staining

Cells were seeded on chamber slides (Ibidi) coated with poly-l-lysine and laminin (see methods for Schwann cell isolation) for 24–48 h, then fixed in 4% paraformaldehyde (EM Sciences) for 10 min at RT, permeabilized in 0.1% bovine serum albumin + 0.2% Triton X-100 for 30 min at RT, then blocked in PBS + 5% goat serum + 5% glycerol + 1% fish gelatin (all from Sigma) for 1 h at RT. Cells were incubated overnight at 4 °C in blocking buffer + rabbit anti-GFAP (Agilent Z0311), washed 3 × 5 min with PBS, then stained with Phalloidin AF594 (Thermo A12381) and anti-rabbit IgG AF488 (Thermo A11008) for 1 h at RT in the dark. Slides were then washed as above and mounted using Prolong Gold with DAPI (Thermo). DAPI, AlexaFluor488, and AlexaFluor594 were imaged with a quad excitation filter (395/476/544/640), dichroic LP beamsplitters (380/505/570), and emission filters (420LP/510-530/590-650). Images were acquired on a Nikon TiE Inverted microscope equipped with an Andor Zyla 5.5MP monochrome CMOS camera using a 20× objective and Nikon Elements acquisition software. Image resolution is 2560 × 2160 pixels (216 nm/px). Quantification of nuclei was performed using Fiji software^79^.

### EM fixation and imaging

Porcine saphenous nerve and optic nerve were isolated immediately upon euthanasia and incubated for 24–48 h at 4 °C in Karnovsky’s fixation solution (4% paraformaldehyde and 3.2% glutaraldehyde in 0.1 M phosphate buffer, pH 7.4–7.6), then transferred to sodium cacodylate buffer (EM Sciences). Tissue was blocked, osmicated, dehydrated, and embedded in LX 112 (EMS) and processed for routine semi-thin and ultrathin sectioning. Sections were stained in uranyl acetate and lead citrate and viewed on a Hitachi Model H-7600 microscope.

### Blood pressure measurements

Pigs were sedated with 5.5 mg kg^−1^ Telazol and blood pressure measurements were taken by automated cuff (BP Accu-Gard, Vmed Technology) and Doppler (ES-100VX MINIDOP, KOVEN Hadeco).

### Statistics

All statistical analysis was done using a Student’s *t*-test to generate two-tailed *p* values using GraphPad software or in R (version 3.43.1) R Studio PKNCA package (version 0.8.1)^77^.

## Electronic supplementary material

Supplementary Information

## Data Availability

The datasets generated during and/or analyzed during the current study are available from the corresponding author on reasonable request. Animals and cell lines generated in this publication are to be distributed by Recombinetics, a for-profit company.
