# Supplementary material for: Genetically engineered minipigs model the major clinical features of human neurofibromatosis type 1
Source: Commun Biol. 2018 Oct 2;1:158. doi: 10.1038/s42003-018-0163-y (PMC6168575; doi:10.1038/s42003-018-0163-y)
Supplement: Supplementary file 1 — Supplementary Information [file 42003_2018_163_MOESM1_ESM.pdf]

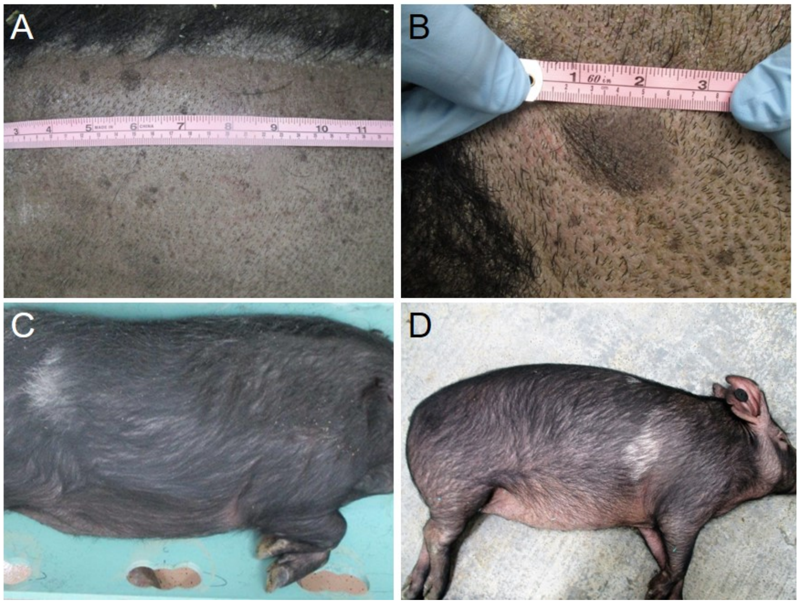


**Supplementary Figure 1. NF1 minipigs display freckling and hypopigmentation.** A. An NF1 F0 minipig at 12 months of age shows freckling along its side and flank. B. An NF1 F1 minipig at eight months of age shows intertriginous freckling and a large CALM. C. An NF1 F0 minipig at eight months of age shows a region of hypopigmented hair that was observed at birth. D. An NF1 F1 minipig at four months of age shows a region of hypopigmented hair that was observed at birth and a large CALM dorsal to region of hypopigmentation that extends across its back.

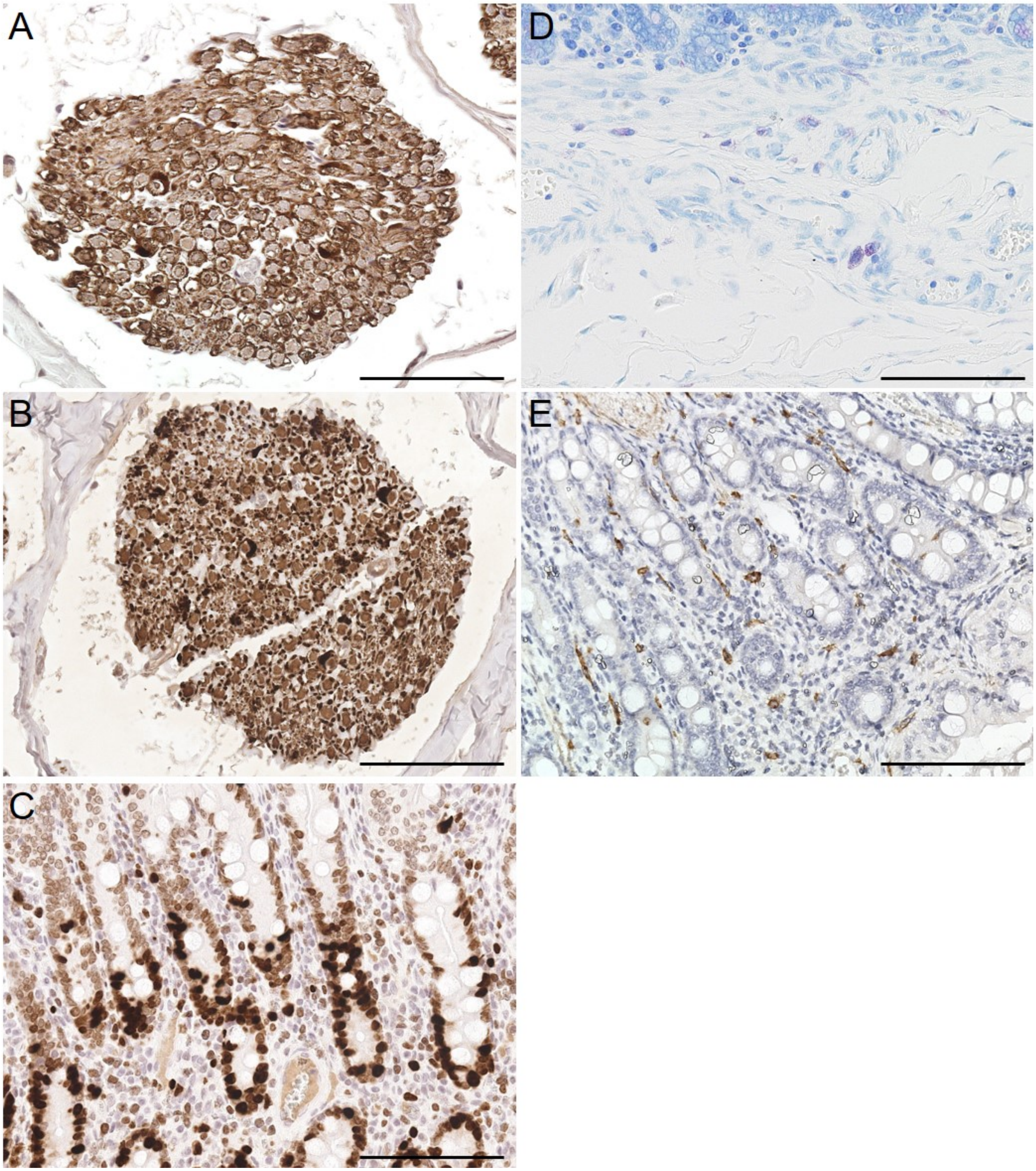


**Supplementary Figure 2. Immunohistochemistry controls from wild-type tissues.** A. Sciatic nerve, S100 β. B. Sciatic nerve, GFAP. C. Small intestine, Ki67. D. Small intestine, Toluidine blue staining. E. Small intestine, c-Kit. Scale bars, 100 μM.

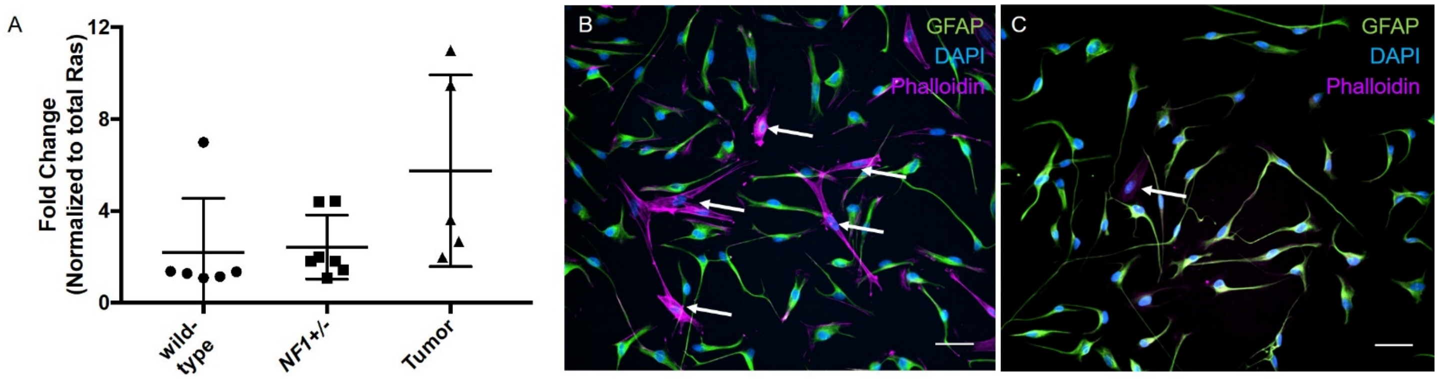


**Supplementary Figure 3. A subset of Schwann cells isolated from NF1 minipig tumors show Ras hyperactivation.** A. Relative levels of Ras-GTP in primary Schwann cells isolated from sciatic nerve (wild-type and *NF1*$^{+/-}$) or neurofibroma (tumor), expressed as a fold change from serum starved to stimulated (5 min). A summary of six independent experiments is shown. Lines represent mean and standard deviation. B. Representative immunohistochemical image from primary minipig Schwann cell lines at passage four, demonstrating GFAP expression (green), counter-stained with DAPI (blue) and phalloidin (magenta). Schwann cell cultures are contaminated with fibroblasts (arrows). C. By passage 8, Schwann cell cultures are >90% pure. Scale bars, 50 μM.

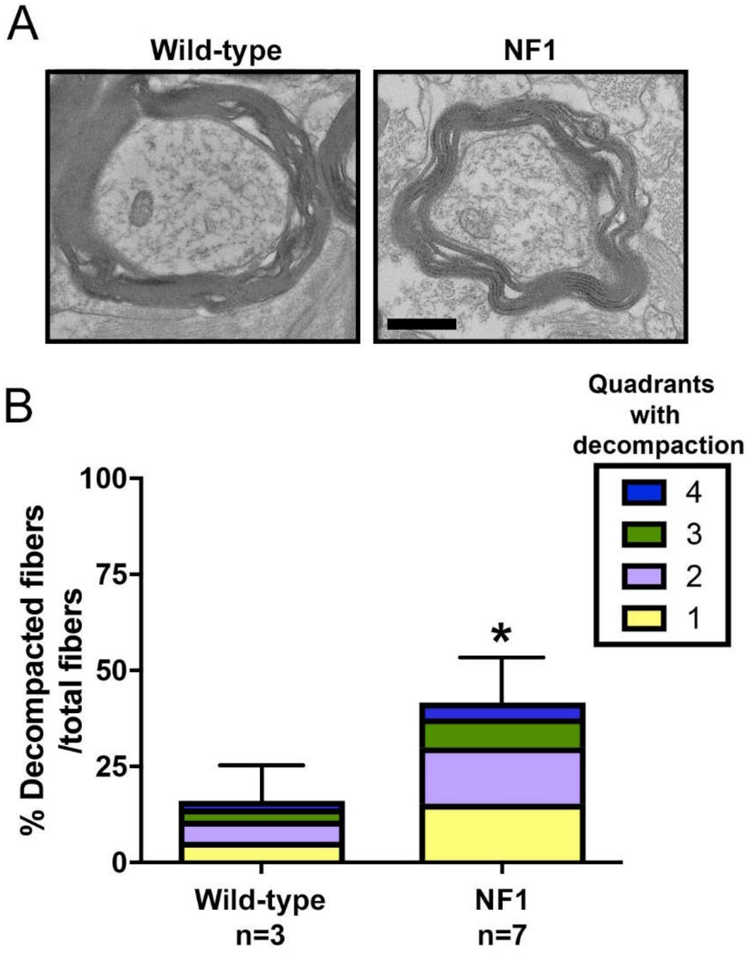


**Supplementary Figure 4. NF1 minipigs show white matter decompaction.** A. Representative electron microscopy images of wild-type and NF1 minipig optic nerve demonstrate myelin decompaction of the optic nerve in NF1 minipigs. Scale bar, 1 µM. B. Gradient bar graph quantifying percent of decompaction shows that NF1 minipigs have a statistically significant increase in white matter decompaction. Error bars represent standard deviation; * P=0.0107.

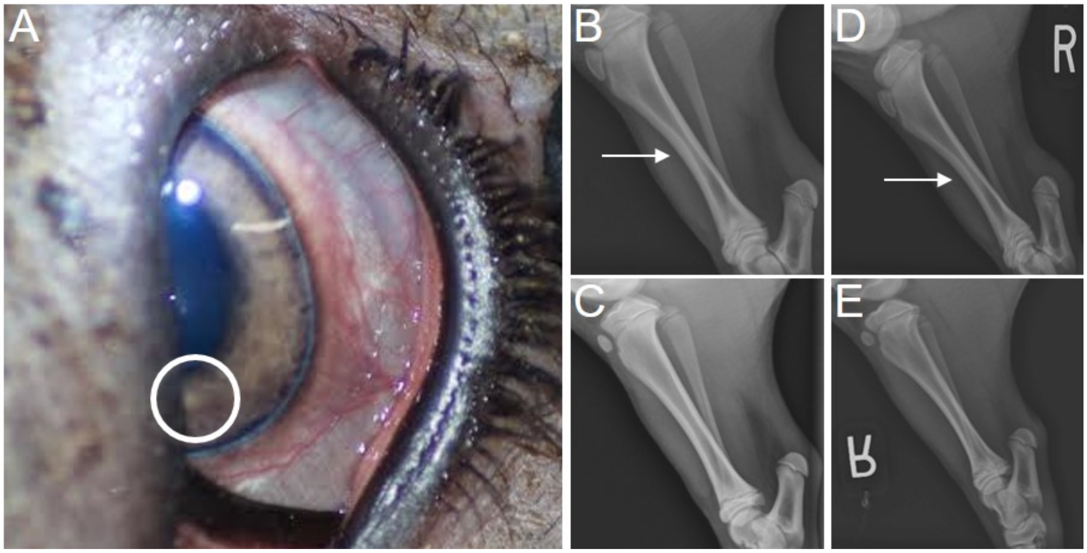


**Supplementary Figure 5. NF1 minipigs show Lisch nodules and skeletal abnormalities.** A. A representative Lisch nodule observed in an NF1 minipig upon slit lamp examination. B. Left pelvic limb X-ray in a pre-pubescent NF1 minipig showing a narrowed tibial diaphysis (white arrow). C. Left pelvic limb X-ray of a pre-pubescent NF1 minipig representative of a normal tibial diaphysis. D. Right pelvic limb X-ray of a pre-pubescent NF1 minipig showing a narrowed tibial diaphysis (white arrow). E. Right pelvic limb X-ray of a pre-pubescent NF1 minipig representative of a normal tibial diaphysis.

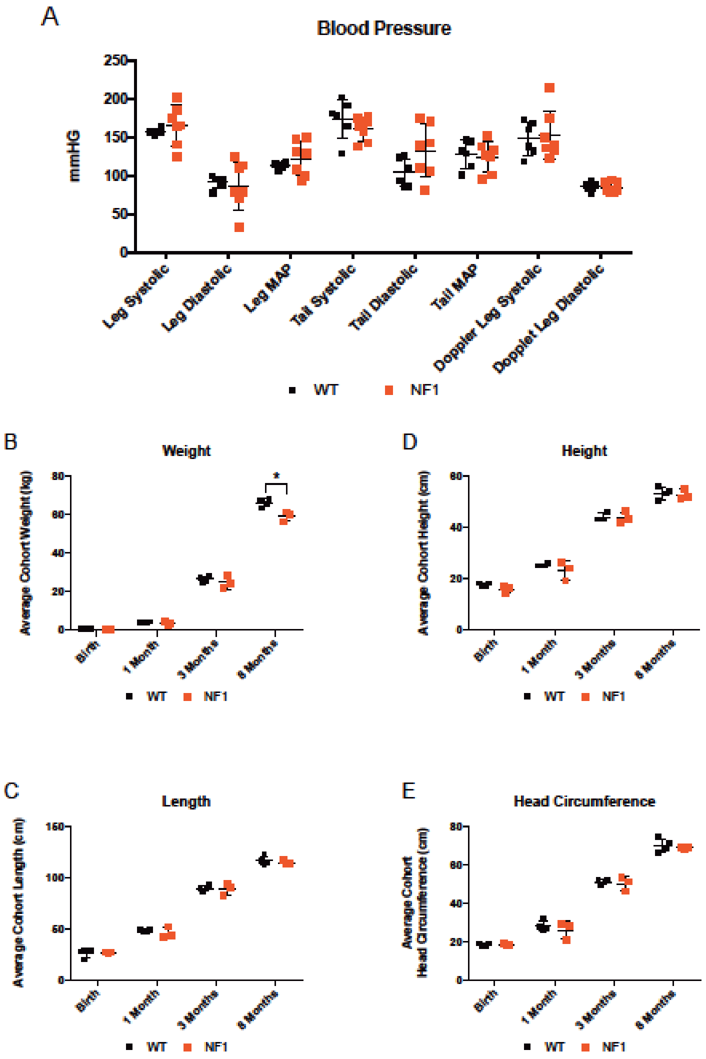


**Supplementary Figure 6. Blood pressure and body measurements in NF1 and wild-type minipigs.** A. Blood pressure was measured in age-matched NF1 (n=7) and wild-type (n=8) minipigs at 8-11 months. Blood pressure was measured by a standard cuff on the front right leg (leg systolic, leg diastolic, leg mean arterial pressure (MAP)) and the tail (tail systolic, tail diastolic, tail MAP), as well as by Doppler on the front right leg (Doppler leg systolic, Doppler leg diastolic). B. Body weight measurements over time. *At 8 months of age NF1 (n=3) minipigs weighed significantly less than their wild-type (n=4) siblings (66.15 ± 2.04 kg versus 59.27 ± 2.58 kg, two-tailed p-value= 0.0106, Students T-Test). C. Body length (snout to tail) measurements over time. D. Body height (shoulder to hoof) measurements over time. E. Head circumference (under chin and behind ears) measurements over time. Error bars represent standard deviation.

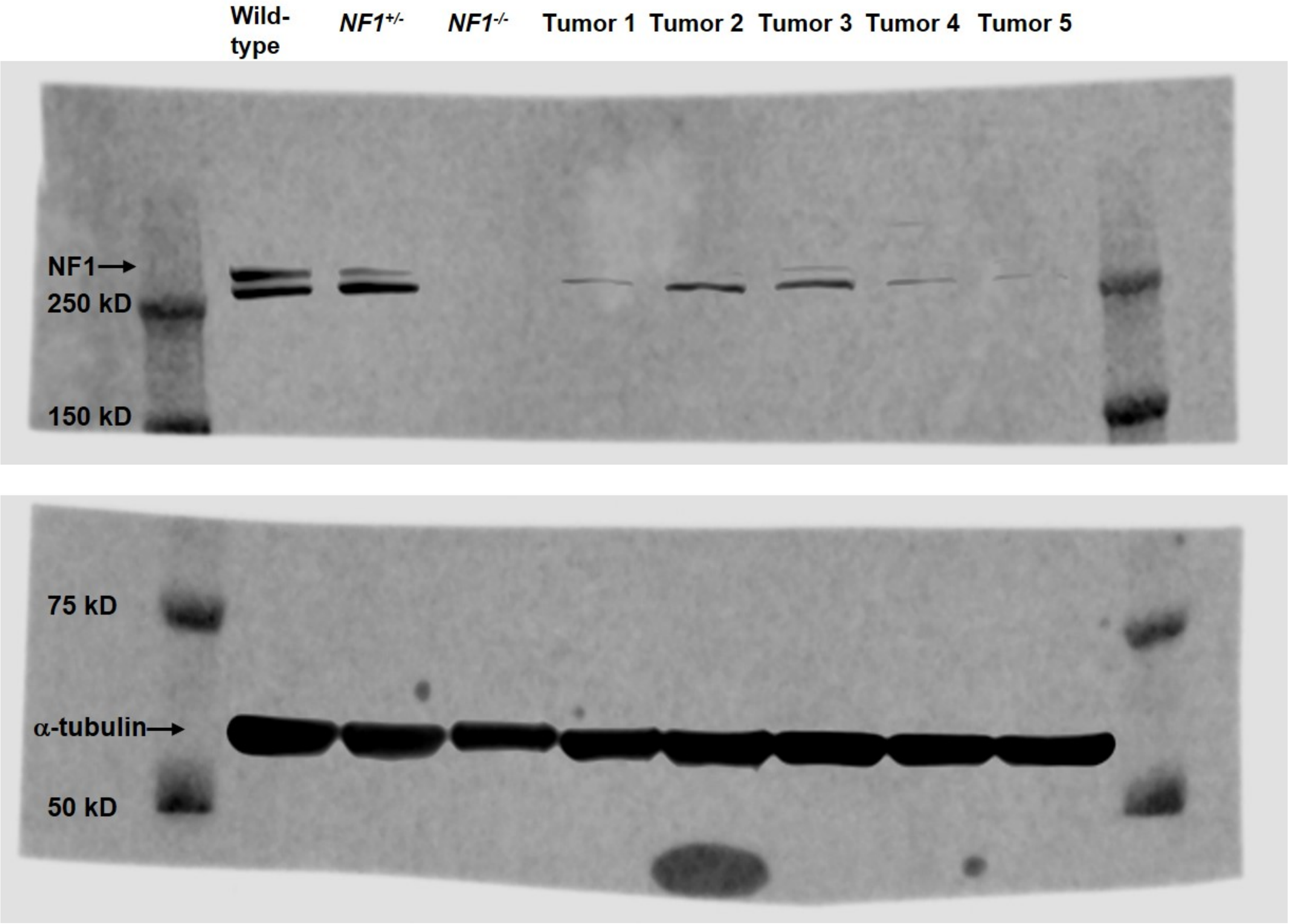


**Supplementary Figure 7. Full length Western blot of Neurofibromin expression in Schwann cell lines.** Wild-type, Schwann cells from wild-type minipig sciatic nerve; *NF1*$^{+/-}$, Schwann cells from NF1 minipig sciatic nerve; *NF1*$^{-/-}$, immortalized human *NF1*$^{-/-}$ Schwann cell line; Tumor 1-5, Schwann cells from NF1 minipig neurofibromas with LOH.

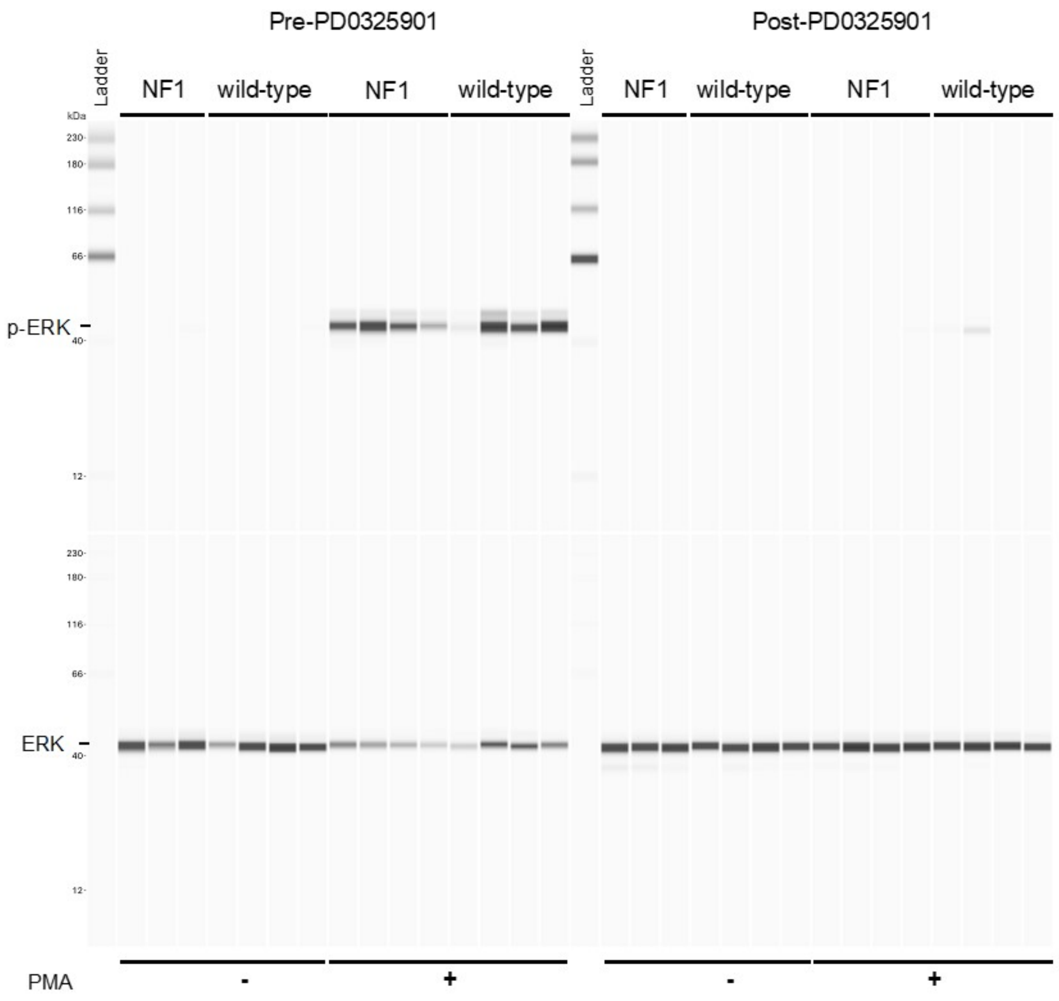


**Supplementary Figure 8. Uncropped capillary electrophoresis images of p-ERK and ERK expression in PBMCs isolated from wild-type and NF1 minipigs before and after treatment with PD0325901.** PMA, phorbol-myristate acid.

| Mutation: NF1:c.5839C>T; p.Arg1947Ter | | | |
|---|---|---|---|
| Age Group (Years) | ≤8 | 9-18 | ≥19 |
| Total Number of Probands | 30 | 8 | 9 |
| Age Range (Years) | 0.25-6.3 | 9.3-17.5 | 22.5-51 |
| Female:Male Ratio | 13:17 | 4:4 | 5:4 |
| Sporadic: Familial: Family History Unknown | 17:4:9 | 5:2:1 | 3:1:5 |
| Fulfilling NIH criteria | 21/30 | 7/8 | 9/9 |
| | | | |
| >5 CALMs | 25/30 | 7/8 | 9/9 |
| Freckling | 18/30 | 6/8 | 8/9 |
| Lisch Nodules | 0/16 | 3/6 | 2/4 |
| Skeletal Abnormalities | 2/30 (pectus excavatum; pseudarthrosis) | 2/8 (non-ossifying fibroma in distal femoral metaphysis & prox. tibial metaphysical regions and scoliosis; long bone dysplasia) | 2/9 (scoliosis; sphenoid wing dysplasia) |
| | | | |
| Externally Visible Plexiform Neurofibromas | 3/27 (trunk, L foot, head) | 1/8 (head) | 1/9 (neck, trunk, L arm) |
| Cutaneous Neurofibromas (cNFs) | 1/30 (2-6 cNFs) | 3/8 | 7/9 |
| Subdermal Neurofibromas | 2/30 | 1/8 | 3/9 |
| Symptomatic Spinal Neurofibromas | 0/30 | 0/8 | 0/9 |
| Asymptomatic Spinal NFs by MRI | 1/1 | 2/2 (bilateral L1-5; sacrum nerve roots) | 0/1 |
| | | | |
| Symptomatic OPG | 0/30 | 0/8 | 0/9 |
| Asymptomatic OPG by MRI | 1/1 | 1/5 | 1/2 |
| Other Neoplasms | 0/30 | 1/8 (lymphangioma on L side of face) | 1/9 (neurofibrosarcoma) |

**Supplementary Table 1. Aggregated phenotypic data of patients with *NF1*$^{R1947*}$ mutations.** Data was collected using a phenotypic checklist on 8,100 (± 8) unrelated, mutation positive NF1 patients[1]. The prevalence of the *NF1*$^{R1947*}$ mutation was 0.75%. Patients were analyzed for a variety of phenotypes and categorized based on age group (≤8 years old, 9-18 years old, and over 18 years old). In addition to the phenotypes described in the table, a subset of patients exhibited learning disabilities and developmental delays, including a 28-year old female with visual/spatial coordination deficits, a 4-year old female with speech delays, a 3-year old male with delayed development, a 2-year old with delayed speech and delayed gross and fine motor skills, and a 4-year old male with impaired speech, impaired motor skills, behavioral and social problems.

| | Left TALEN | Spacer | Right TALEN | |
|---|---|---|---|---|
| *NF1* TALEN | GATGATGCCAAACGAC | aaagagttactgcgat | CCTTGATAAGCTGATA | |
| | **Sequence** | | | |
| *NF1* HDR oligo | tcaaatctagtacgtttttgtaagcacaatgatgatgccaaaTgaAGCTTcaaagagttactgcgatccttgataagctgataacaatga | | | |
| | **Left Primer Sequence** | **Right Primer Sequence** | **Annealing Temp.** | **Amplicon Size** |
| *NF1* R1947X Primers | CCTGCCCCCACCATCTTCTTATT | GCTCTCGTACAGTGCTTTGCACAA | 60 | 365 bp |

**Supplementary Table 2. TALEN and primer design.** TALENs were designed to bind within swine *NF1* exon 41, near $NF1^{R1947}$. The sequence of the binding site for the left TALEN with 16 repeat variable dinucleotides (RVDs) and the right TALEN with 16 RVDs are shown, along with the 16-base pair (bp) spacer sequence. The *NF1* HDR oligonucleotide sequence is shown with homology to the wild-type *NF1* gene (lower case letters). Nucleotides that were added or changed are capitalized and create an $NF1^{R1947*}$ mutation (red) and a novel *Hin*dIII RFLP site (underlined). Primer sequences are shown for primers designed to amplify the region around $NF1^{R1947}$.

| Genotype | Dose (mg $kg^{-1}$) | $C_{max}$ (ng $mL^{-1}$) | $t_{max}$ (hr) | $t_{1/2}$ (hr) | $AUC_{0\rightarrow48}$ (ng $mL^{-1}$ $hr^{-1}$) |
|---|---|---|---|---|---|
| Wild-type | 0.79 | 120±62 | 5±5 | 11±2 | 1130±430 |
| NF1 | 0.79 | 131±35 | 5±3 | 9±2 | 1665±244 |

**Supplementary Table 3. Pharmacokinetic parameters**. $C_{max}$ = maximum concentration (ng $mL^{-1}$), $t_{max}$ = time at maximum concentration (hours), $t_{1/2}$ = half-life (hours), $AUC_{0\rightarrow48}$ = area under the plasma drug concentration-time curve from 0 to 48 hours (mL $hr^{-1}$). Mean with standard deviation measurements.

## Supplementary References:


1. Koczkowska, M. *et al.* Genotype-Phenotype Correlation in NF1: Evidence for a More Severe Phenotype Associated with Missense Mutations Affecting NF1 Codons 844-848. *American journal of human genetics* **102**, 69-87, doi:10.1016/j.ajhg.2017.12.001 (2018).
